# A synthetic method for preparing double channelling materials, and an operational mechanism for selective p- and n-type channels for gas sensing

**DOI:** 10.1038/s41378-026-01253-w

**Published:** 2026-05-06

**Authors:** Myung Sik Choi, Han Gil Na, Jeong Yun Hwang, Seung Yong Lee, Sanghyun Ji, Jimyeong Park, Sun-Woo Choi, Kyu Hyoung Lee, Changhyun Jin

**Affiliations:** 1 Department of Nano & Advanced Materials Science and Engineering, Kyungpook National University, Sangju, Republic of Korea; 2 UDerive, Incheon, Republic of Korea; 3 Department of Materials Science and Engineering, Yonsei University, Seoul, Republic of Korea; 4 Division of Advanced Materials Engineering Kongju National University Cheonan, Cheonan, Republic of Korea; 5 Department of Materials Science and Engineering, Kangwon National University, Samcheok, Republic of Korea

**Keywords:** Materials science, Electrical and electronic engineering

## Abstract

A new synthetic strategy and associated mechanism have been developed, in which two carrier conduction channels of n- and p-type semiconductors on the surface of one material are automatically and advantageously selected during surface reactivity. The key step is to uniformly channel non-equilibrium metal oxides of CuO_x_ and SnO_x_ throughout the sample by applying a flame chemical vapour deposition technique for 5 s. Unlike the original SnO_2_ semiconductor and Cu metal, the resulting material possessed intermediate physicochemical properties. It has been demonstrated that an oxidising gas, NO_2_, and reducing gas, H_2_S, can be alternately adsorbed, which was facilitated by the automatic selection of p- or n-type channels. This solid-solution sensing method utilizing non-equilibrium compositions can be employed in other applications involving semiconducting metal oxide gas sensing, even at low temperatures.

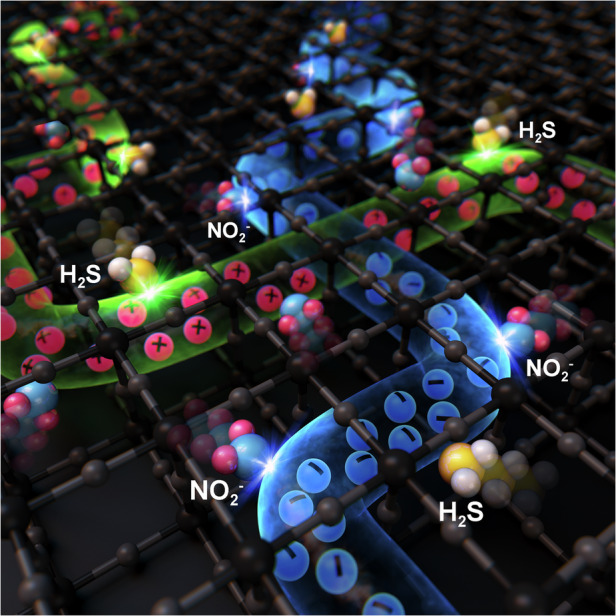

## Introduction

Semiconductor-based gas sensors are the key to how carriers such as electrons and holes can change current or resistance upon the adsorption/desorption of oxidising or reducing gases onto n- or p-type semiconductors^1–12^. As such, four different combinations of semiconductors can exist: n-type/oxidising gas, n-type/reducing gas, p-type/oxidising gas, and p-type/reducing gas. These combinations can be classified into representative gas sensing indices that are affected by both the process temperature and gas concentration^13–18^. That is, through experimental or empirical data, it may be possible to sequentially arrange specific semiconductor-reactive gas pairs that show exceptionally good gas sensing characteristics. For example, SnO_2_, which is a typical n-type semiconductor, demonstrates a much higher response to oxidising gases when oxidising or reducing gases are present under the same process conditions and gas concentrations^19–21^. Therefore, in the presence of oxidising gases, SnO_2_ is naturally more likely to be used at lower process temperatures and gas concentrations than reducing gases^22,23^. Similarly, CuO, which is a p-type semiconductor, is more suitable for the adsorption of reducing gases at lower process temperatures and gas concentrations when exposed to oxidising or reducing gases^24–28^. However, because there are many exceptions from this straightforward approach^29–32^, it cannot be established as an absolute law or theory. Despite this, SnO_2_ and CuO may be regarded as reference materials as they exhibit distinct differences in how they react with oxidising or reducing gases. In the case of SnO_2_ selectivity, the mechanism for oxidising gas adsorption has not been determined. However, it has been reported to show the best sensing characteristics in NO_2_^33–35^. On the other hand, CuO is known to show the best sensing characteristics in H_2_S through comparative analyses with other reducing gases^36–38^. As a result of this excellent selectivity for a particular gas, one-to-one matching of the semiconductor-reactive gas is achievable. However, until now, the most logical approach and choice of semiconducting materials for gas sensing could not be selected until a clear understanding of the identity of the reaction gas was obtained, which has many associated limitations in terms of unconditional application in industry. In other words, since SnO_2_ is selective for sensing NO_2_ and CuO is selective for H_2_S, as described above, the type of semiconductor applied for each gas must be alternated to achieve optimal sensing efficiency. Therefore, we sought to utilize a single material containing both semiconductors to detect oxidising or reducing gases, rather than using a different material for each gas. In other words, we sought to develop a single material containing both SnO_2_ and CuO to detect NO_2_ or H_2_S separately, rather than together. For successful execution of this concept, selective adsorption must occur between the gas and either the n- or p-type semiconductor within the double channel material. In this study, molecular dispersions were uniformly constructed into SnO_x_ and CuO_x_ semiconductors through flame chemical vapour deposition (FCVD) for a duration of 5 s, which is a surface modification process developed previously in our lab. Here, the SnO_x_ channels were activated upon the adsorption of the NO_2_ oxidising gas, while the CuO_x_ channels were activated upon the adsorption of the H_2_S reducing gas. Unlike conventional semiconductor junctions, where a depletion layer is generally formed in a PN junction, the proposed structure is a double-channelling configuration, in which the gas and sample operate via NN + PN and PP junctions. Specifically, FCVD yields two types of semiconductors containing a non-equilibrium oxide (e.g. SnO_x_ and CuO_x_) co-existing as a solid-solution of SnO_x_-CuO_x_ by simultaneously oxidising (for Cu) and reducing (for SnO_2_) a nanocomposite of Cu and SnO_2_ bilayers. Overall, the core concepts demonstrated here-in encompass the development and application of a synthetic process for the fabrication of a multi-purpose gas sensing device, which may pave the way to a plethora of new sensing applications.

## Experimental methods

Au nanoparticles (3 nm) were deposited onto an alumina substrate through direct current (DC) sputtering. The DC sputtering conditions were as follows: Process current = 10 mA; process time = 1 minute; vacuum condition = 2 × 10^–4^ mTorr; working gas = Ar; process temperature = room temperature (RT (25 °C)). Next, Sn powder (1 g) was put into an alumina boat with the alumina substrate containing deposited Au placed upside down to directly face the Sn powder, and SnO_2_ nanowires were grown by thermal evaporation. The conditions for thermal evaporation were as follows: Process temperature = 900 °C; rate of temperature increase = 10 °C·min^−1^; process time = 1 h; reactive gas = oxygen:argon in a 1:9 ratio; gas pressure = 2 Torr. Thereafter, Cu was deposited (thickness = 3 nm) through DC sputtering to form a layer of Cu coating the SnO_2_ nanowires. The conditions for DC sputtering were as follows: Process current = 10 mA; process time = 1 min; vacuum condition = 2 × 10^–4^ mTorr; working gas = Ar; process temperature = RT (25 °C). Finally, flame chemical vapor deposition (FCVD) was applied to the core-shell structure for a duration of 5 s.

The morphology, composition, crystallinity, and energy states of the final sample were measured by scanning electron microscopy (SEM; Hitachi S-4200, Hitachi), transmission electron microscopy (TEM; JEM-2100 F, JEOL), energy dispersive X-ray spectroscopy (EDS, JEM-2100 F, JEOL), X-ray photoelectron spectroscopy (XPS; Thermo Fisher Scientific Co.), Raman spectroscopy (LabRAM Hr800, JOBIN YVON, laser wavelength: 355 nm), powder X-ray diffraction (p-XRD, Rigaku Ultima IV diffractometer, X-ray source: Cu K_α_ irradiation (λ = 1.5418 Å)), and ultraviolet photoelectron spectroscopy (UPS; Thermo Fisher Scientific Co.).

A sputtering mask was placed on the final sample formed on the alumina substrate, and a 50/300 nm Ti/Au bi-layer electrode was deposited using a DC sputterer. The electrode width of the sputter mask was 600 µm, and the spacing between the electrodes was 400 µm. Sputtering conditions were 80 mA for 12 minutes for a 300 nm thick Au deposition. The sensor element on which the electrodes were deposited was placed in a sensor chamber equipped with a ceramic heater that can be controlled up to 300 °C. And the change in resistance was measured using a multimeter (Keithley 2450). Target gases were injected into the chamber, which induced a response in the sensor element, and no additional pump was used to exhaust it. All target gases were purchased and used in gas cylinders containing 100 ppm of toxic gas in N_2_ base. The concentration of the target gas was controlled using air gas with a relative humidity (RH) of 0% using a mass flow controller (MFC, Kofloc 3660), and the total flow rate was adjusted to 500 standard cubic centimeters per minute (sccm). The target gas was completely mixed with a controlled concentration using an MFC via a static mixer just before injection into the sensor chamber. The temperature and humidity of the target gas were maintained at constant RH and room temperature using a thermohygrometer (Rotronic HF532). The adsorption reaction with the target gas was confirmed by injecting toxic gases with controlled concentrations (2, 6, 10 ppm) for 500 s, and the desorption reaction with the target gas was confirmed by injecting pure air gas for 1000 s. The ratio of resistance when air (R_a_) and the target gas (R_g_) were introduced was measured through a home-made gas sensor. In this study, in the low temperature range (25 °C and 100 °C) rather than the high temperature range (300°C), when an oxidizing gas such as NO_2_ is used, n-type SnO_x_ is activated, and when a reducing gas such as H_2_S is used, p-type CuO_x_ is activated. Therefore, the response is expressed as R_g_/R_a_ regardless of the type of target gas. The starting point of the saturation region of the dynamic sensing curve was determined as the point at which the dynamic curve reached 90% regardless of the response and recovery curves.

## Results and discussion

### Synthesis of a solid-solution composed of SnO_x_ and CuO_x_

**Fig. 1:**
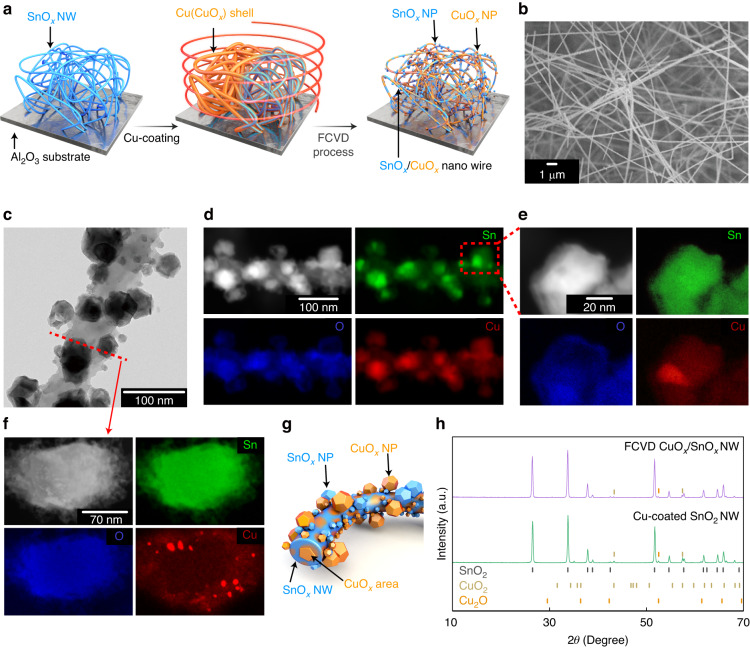
Morphologies and compositions of SnO_x_-CuO_x_ solid-solution samples. **a** Schematic diagram showing the SnO_x_-CuO_x_ solid-solution formation process. **b** SEM image of typical SnO_2_/Cu core-shell structures. **c** Typical TEM image of a SnO_x_-CuO_x_ solid-solution containing two compositions of SnO_x_ and CuO_x_ following the FCVD process. **d** Mapping of each constituent element distributed throughout the sample by composition. **e** Mapping of each constituent element distributed in the local area by composition. **f** Components in the vertical plane of the SnO_x_-CuO_x_ nanowires. **g** Schematic diagram showing two channels of SnO_x_ and CuO_x_ spread throughout the sample. **h** Powder XRD spectra before and after FCVD

Regardless of the oxidising and reducing gases, in order to form a useful device that is comprised of dual channels of n- and p-type semiconductors, SnO_x_ and CuO_x_ must form a uniform solid-solution^39^. To enable this unique double channelling, as seen in Fig. 1a, the high-temperature thermal energy from FCVD was injected instantaneously onto the surface of the sample following core-shell sputtering of Cu over SnO_2_ nanowires, resulting in a compositional change from a double layer of SnO_2_ and Cu to a single layer component of SnO_x_-CuO_x_ in which SnO_x_ and CuO_x_ were uniformly distributed. This step is the most essential feature of this synthetic process, and the advantages provided in terms of precursor, equipment, pre- and post-treatment, temperature, time, and vacuum have been reported elsewhere^40^. Thus, FCVD may be applied for a wide variety of purposes and effects regardless of the material type, composition, and reaction conditions. The samples must be composed of SnO_x_ and CuO_x_ components that can exhibit semiconductor properties, which may be achieved through strict control of the process parameters during FCVD (Supporting Information, (SI) Fig. S1). The competitive characteristics of the samples in which SnO_x_ and CuO_x_ were uniformly distributed can be summarised as follows:

1. Channels were formed evenly throughout the sample in the form of a non-equilibrium oxides combined with oxygen.
2. The final sample can be regarded as a configuration in which both SnO_x_ and CuO_x_ take an intermediate form of semiconductor and metal, unlike the description of the SnO_2_ semiconductor core and Cu metal shell.
3. If, through the injection of energy, only the Cu shell reacts and is aggregated in the form of CuO_x_ islands, and CuO_x_ channelling is not achieved on the entire sample, the sample may not exhibit gas sensing characteristics of the CuO_x_ base. However, the strong energy from FCVD can simultaneously carry out oxidation and reduction of the core and shell.

One of the greatest advantages of FCVD is that the function and efficiency of gas adsorption on the sample surface are significantly enhanced, even though the process is only applied for less than 5 s. However, unlike in other FCVD applications^40^, to achieve the two-channel objective, various n-type and p-type semiconductors - ranging from SnO_2_ to SnO_x_, SnO, CuO, Cu_2_O, and CuO_x_ - must be able to penetrate the entire sample. Furthermore, the entire sample must not be uniformly controlled at once; rather, it must be locally and non-uniformly controlled multiple times to form a three-dimensional two-channel structure. SI, Table S1 succinctly summarizes the characteristics of the samples before and after FCVD.

### Morphological, compositional, and microstructural characteristics of the SnO_x_-CuO_x_ solid-solution

The morphologies of the SnO_x_-CuO_x_ solid-solution were analysed by electron microscopy. The scanning electron microscopy (SEM) image of a typical SnO_x_-CuO_x_ core-shell structure with a thickness of ~ 100 nm and a length of several hundred μm is shown in Fig. 1b. The surface of each nanowire was smooth and uniform and in the absence of irregular protrusions, even though they were produced by sputtering, which is a physical deposition method whereby it is more difficult to obtain a homogeneous coating layer than chemical vapour deposition methods, such as metal-organic chemical vapour deposition or atomic layer deposition^41–43^. However, if the composition of the surface was changed from Cu (or CuO_x_) to the SnO_x_-CuO_x_ solid-solution by FCVD, irregular and small dendritic phases would be generated on the surface, resulting in a rough and uneven surface (Figs. 1c and SI and S1). In this way, the newly formed SnO_x_-CuO_x_ solid-solution from the original SnO_2_/Cu double structure can serve to widen the cross-sectional area of the surface, which is anticipated to increase gas response by increasing both the area and time for the reaction gas to react. However, as shown in Fig. 1c, other compositions aside from the SnO_x_-CuO_x_ solid-solution were observed as a result of the thermal energy injected during FCVD, which exceeded the threshold for maintenance of SnO_2_ as an oxide. That is, SnO_2_ decomposed into Sn and O_2_, whereby Sn hardened as a metal^44^. Alternatively, the FCVD process occurred so quickly that complete conversion of Cu into CuO_x_ did not occur; only the outer side was oxidised to CuO_x_ and the inner core remained as metallic Cu^45^. This transmission electron microscopy (TEM) image provided indirect evidence of SnO_2_ collapsing and O_2_ vaporising, and thus a compositional change to SnO_x_ occurred with oxidation of Cu to CuO_x_. The size of the droplet (darker region) formed on the dendritic end was ~20–80 nm in length. As described above, some metal components, such as Sn and Cu, may be present, but most of the thin oxide layer (lighter region) surrounded the droplet. In other words, the oxide layer penetrating the sample can be combined with Cu and Sn to serve as a semiconducting metal oxide throughout the sample. Moreover, considering that FCVD is complete within 5 s, it is more certain that the non-equilibrium metal oxides were evenly distributed because the oxide layers could not match the stoichiometry of the existing Sn and Cu in that timeframe.

The results of component mapping to confirm whether the SnO_2_/Cu composition had changed to the SnO_x_-CuO_x_ solid-solution following FCVD are shown in Fig. 1d–f. It was confirmed that Sn, Cu, and O were evenly distributed in all parts of the sample, even if localised regions were analysed, as shown in Fig. 1e, or if the nanowire was analysed in the direction of maximum thickness, as shown in Figs. 1f. and SI, S2. As mentioned above, it can be assumed that a phase change occurred from the existing SnO_2_/Cu double layer to the composition in which SnO_x_ and CuO_x_ were mixed (Fig. 1g). Here, n-type SnO_x_ and p-type CuO_x_ do not exist as a single ternary material, implying that both channels may be formed, facilitating dual detection of either oxidising or reducing gases. Even if the Sn and Cu composition differed, there is still a possibility that SnO_x_ and CuO_x_ can coexist in any part of the sample.

The powder X-ray diffractograms (p-XRD) are displayed in Fig. 1h and show that SnO_x_ and CuO_x_ coexist simultaneously following FCVD, and the original SnO_2_-core and Cu (CuO_x_ as CuO_2_ or Cu_2_O)-shell do not exactly match. Since p-XRD provides insight into the stable phases present in a sample, and SnO_x_ and CuO_x_ do not exactly match stoichiometrically, they must not represent exact points compared to the reference (e.g., stable phase). Therefore, all diffraction peaks observed in Fig. 1h can be considered slightly shifted from their original point. Such a combination of SnO_x_ and CuO_x_ non-equilibrium phases can be a key cause for two channels in gas sensing, which were not observed previously.

### Surface chemical bonding and energetic properties of the SnO_x_-CuO_x_ solid-solution

**Fig. 2:**
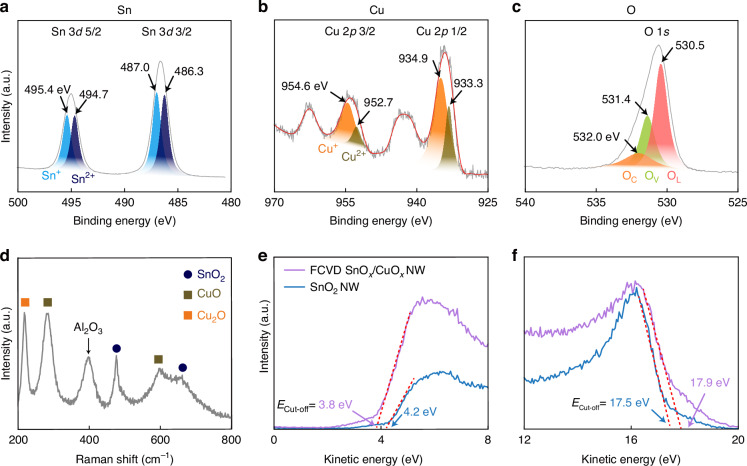
Surface chemical bonding characteristics of SnO_x_-CuO_x_ solid-solution samples. **a**–**c** XPS spectra showing the binding energies of: **a** Sn, **b** Cu, and **c** O, which are the components of the SnO_x_-CuO_x_ solid-solution sample. **d** Raman spectra showing various forms of SnO_x_ and CuO_x_ mixed in the sample. **e** Valence band edges and **f** characteristic E_cut-off_ values of bare SnO_2_ and the SnO_x_-CuO_x_ nanowires

To predict the surface reactivity in the SnO_x_-CuO_x_ solid-solution, measurements by X-ray photoelectron spectroscopy (XPS; Fig. 2a–c), Raman (Fig. 2d), and ultraviolet photoelectron spectroscopy (UPS; Fig. 2e–f) were carried out. Since FCVD was complete within a few s, the resulting chemical bonds and various types of defects on the sample surface can have significant effects on the energetic state of the conduction (or resistance) channel in the sample during gas sensing, which deals with chemical reactions on the instantaneous surface. From the Sn, Cu, and O XPS spectra, which are components of the SnO_x_-CuO_x_ solid-solution, various types of binding energies were observed. First, in the case of Sn (Fig. 2a) following Gaussian fitting, Sn^+^ peaks corresponding to Sn 3*d*_5/2_ and Sn 3*d*_3/2_ were observed at 495.4 eV and 487.0 eV, respectively. Additionally, Sn^2+^ peaks corresponding to Sn 3*d*_5/2_ and Sn 3*d*_3/2_ were present (494.7 eV and 486.3 eV, respectively)^46,47^. A study in which FCVD was performed on bare SnO_2_ nanowires has shown that FCVD can generate peaks of Sn alone, rather than oxides^48^. Therefore, in the present study, the FCVD process was employed to generate sufficient non-equilibrium compositions, as shown in Fig. 1h. Similarly, in the case of Cu, which exhibited two shake-up satellite peaks^49–51^ (Fig. 2b), Cu^+^ peaks corresponding to Cu 2*p*_3/2_ and Cu 2*p*_1/2_ were present at 954.6 eV and 934.9 eV, respectively. In addition, Cu^2+^ peaks corresponding to Cu 2*p*_3/2_ and Cu 2*p*_1/2_ at 952.7 eV and 933.3 eV, respectively, were confirmed^52,53^. In the case of oxygen (Fig. 2c), various binding environments were observed in the O 1 *s* XPS spectrum, such as lattice oxygen (O_L_) at 530.5 eV, oxygen vacancies (O_v_) at 531.4 eV, and chemisorbed oxygen (O_c_) at 532.0 eV^54,55^. Moreover, various CuO_x_ compositions, such as CuO and Cu_2_O, exist alongside the initial core SnO_2_, as confirmed by Raman spectroscopy (Fig. 2d)^56,57^. This confirmed the existence of newly formed CuO, which was not observed by p-XRD. That is, from p-XRD (Fig. 1h), XPS (Fig. 2a–c), and Raman (Fig. 1d), it can be inferred that various CuO_x_ (CuO_2_, Cu_2_O, CuO) channels built upon SnO_x_ (SnO_2_) channels are possible.

As shown in SI, Fig. S3, valence band maxima (VB_max_), Fermi levels (E_f_), and work function (Φ) values have been determined from the energy cut-off (E_cut-off_) value obtained by UPS, which was analysed to determine the change in kinetic energy based on the energy of the incident beam (21.2 eV). The initial SnO_2_ nanowires had a valence band maximum energy of 4.2 eV (Fig. 2e) and a work function energy of 3.8 eV (Fig. 2f; e.g., 21.2 eV − 17.5 eV + 0.1 eV (correction value of the equipment) = 3.8 eV), respectively (SI, Fig. S3a–S3c). However, the valence band maximum energy and work function energy decreased to 3.8 (Figs. 2e) and 3.4 eV (Fig. 2f), respectively (e.g., (21.2 eV − 17.9 eV + 0.1 eV (correction value of the equipment) = 3.4 eV) for the SnO_x_/CuO_x_ solid-solution sample (SI, Fig. S3d–S3f) following FCVD. Usually, changes in Fermi or work function energy in single-channel semiconductors are a result of improvements or a decrease in response during gas sensing^58,59^.

### Oxidising/reducing gas sensing behaviour in SnO_x_-CuO_x_ with double channelling

**Fig. 3:**
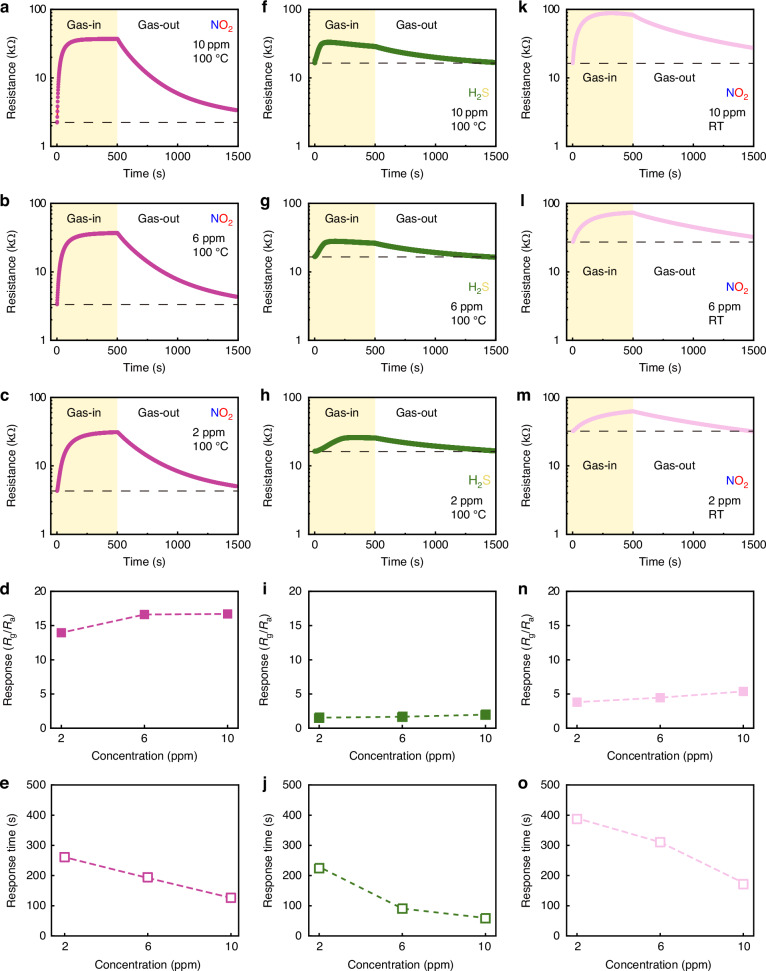
Changes in resistance, response, and response time in SnO_x_-CuO_x_ solid-solution samples at varying concentrations (2–10 ppm) and temperatures (100 °C and 25 °C) of oxidising gas (NO_2_) and reducing gas (H_2_S). **a**–**e** NO_2_ gas at 100 °C, **f**–**j** H_2_S gas at 100 °C, **k**–**o** NO_2_ gas at 25 °C

It is difficult to conclude that the samples synthesised by FCVD have two different semiconducting channels, n-type SnO_x_ and p-type CuO_x_, by the above-described analyses. Therefore, to confirm the possibility that both channels can be activated, the change in the carrier width in the sample was examined while adsorbing both the oxidising NO_2_ and reducing H_2_S gases. The basic mechanism of gas sensing is that two types of gas do not directly exchange carriers with the sample surface, but the reaction always proceeds through the oxygen surrounding the sample surface, regardless of whether the gas to be reacted (adsorbed) is an oxidising or reducing gas^60–62^. That is, the reacting gas reacts with oxygen first, and the oxygen affected by this reaction then reacts again with the sample. In other words, the reaction is chained and sequentially proceeds using oxygen as an intermediate medium. First, when NO_2_ oxidising gas is introduced (Fig. 3a–e and Fig. 3k–o), n-type SnO_x_, which is more selective for NO_2_ than CuO_x_, detects the gas and shows a change in resistance. Currently, if the n-type SnO_x_ forms a channel, the oxidising gas serves to remove the electrons on the sample surface, eventually reducing the electron carrier channelling width within the sample and increasing resistance. At 100°C, the responses (Fig. 3a–d) of the gas sensing based on NO_2_ concentration (10 ppm, 6 ppm, 2 ppm) were 16.7, 16.62, and 13.96, respectively, and the response time (Fig. 3e) was the shortest (126 s) at a concentration of 10 ppm (Fig. 3e), as described in SI, Table S2. This is a superior result even compared to bare SnO_2_ (e.g., not a solid-solution; Table 1)^63–69^. While most gas sensing exhibits no response in the low temperature range (e.g. RT–100 °C)^70,71^, the capability of detecting NO_2_ at RT (25 °C) with responses of 5.38, 4.47, and 3.82 at 10, 6, and 2 ppm NO_2_ (Fig. 3k–o) while maintaining the double channelling structure proves the superiority of this structure relative to other gas sensing devices (SI, Fig. S4).

**Table 1:** Comparison of SnO_2_-based gas sensors for NO_2_ gas

Materials	NO_2_ gas conc. (ppm)	Temperature (°C)	Response (R_g_/R_a_)	Response time (*s*)	Reference
SnO_x_	10	100	16.7	126	This work
Bare SnO_2_	10	100	~4	240	^63^
SnO_2_ thin film	100	200	19	7	^64^
SnO_2_ nanowire	2	150	14	292	^65^
SnO_2_ film	10	260	20.3	6	^66^
SnO_2_ single layer	1	150	10.2	130	^67^
SnO_2_ nanobelt	50	200	~67	<15	^68^
SnO_2_ nanorod	100	300	3.1	150	^69^

Apart from NO_2_, when a reducing gas, such as H_2_S, was introduced (Fig. 3f–j), p-type CuO_x_, which shows greater selectivity for H_2_S than n-type SnO_x_, detected the gas and showed a change in resistance. At this time, if n-type SnO_x_ reacted with the reducing gas of H_2_S, the resistance curves in Fig. 3f–h would not have increased, but rather, the resistance would have decreased. However, it was confirmed that the p-type CuO_x_ reacted with a higher resistance to adsorption of H_2_S with responses of 1.99, 1.68, and 1.55 to 10, 6, and 2 ppm H_2_S, respectively (SI, Table S2). The high resistance to adsorption of H_2_S is a clear demonstration that a reaction occurred with the p-type CuO_x_. Similarly to SnO_x_, if the p-type CuO_x_ forms a channel, the reducing gas serves to supply electrons on the sample surface, which in turn reduces the contribution of holes in the p-type semiconductor. Therefore, this reduction of carriers at the surface eventually narrows the width of the hole carrier channelling inside the sample so that the resistance increased as a combination between n-type semiconductor and oxidising gas. At this time, the results obtained in terms of change in resistance based on H_2_S concentration (10 ppm, 6 ppm, 2 ppm) at 100 °C demonstrate the superior gas sensing performance, even compared to bare CuO (Table 2)^72–78^. Despite the formation of a double channelling structure, the main reason for the absence of any loss in gas sensing capabilities in comparison to a single channel structure may be attributed to the use of FCVD for sample preparation. This result demonstrates the ability to exhibit amphoteric behaviour in one sample in which both n- and p-type semiconductors exist separately, as opposed to a single compound, such as SnCuO_x_. However, porous CuO nanosheets^79^ or leaflet-like CuO nanosheets^80^, which can strategically provide numerous gas adsorption sites, perform much better than our sample in terms of sensing temperature and sensitivity. Additionally, as shown in SI, Table S3, some of the gas-sensing characteristics of our sample may be inferior to those of other SnO_2_-CuO heterojunctions. However, the two-channel synthesis method through solid-solution within s and the low-temperature sensing capability afforded by the increase in cross-sectional area are sufficiently useful from an engineering perspective. In fact, our sample does not sense all gases at all temperatures. Gas sensing occurs only at 100 °C for both NO_2_ and H_2_S. Despite two channels operating simultaneously, the selectivity is excellent, as no other gases are sensed at a temperature of 100 °C. Additionally, in low-concentration NO_2_ gas (SI, Fig. S5a–S5d), the reliability evaluation of the SnO_x_-CuO_x_ solid-solution gas sensor yielded stable results across all tests, including a low concentration range (0.2–1 ppm, SI, Fig. S5a), a low limit of detection (0.15 ppm, SI, Fig. S5b), a wide relative humidity range (0–80%, SI, Fig. S5c), and strong repeatability (eight times, SI, Fig. S5d). However, in low-concentration H_2_S gas (SI, Fig. S5e and S5f), the probability of reducing the hole due to adsorption in the p-type channel is low, and the offsetting effect due to adsorption in the n-type channel is also partially observed. Hence, the sensing performance was barely noticeable.

**Table 2:** Comparison of CuO-based gas sensors for H_2_S gas

Materials	H_2_S gas conc. (ppm)	Temperature (°C)	Response (R_g_/R_a_ or R_a_/R_g_)	Response time (*s*)	Reference
CuO_x_	10	100	1.99	59	This work
CuO thin film	10	100	0.15	110	^72^
CuO nanoparticles	10	80	0.098	20.4	^73^
CuO nanoneedle arrays	10	150	0.8	98	^74^
CuO thin film	100	200	0.25	80	^75^
CuO nanorod	100	300	4	200	^76^
CuO nanorod	5	150	2.4	300	^77^
CuO nanowire	100	300	1.861	>500	^78^

**Fig. 4:**
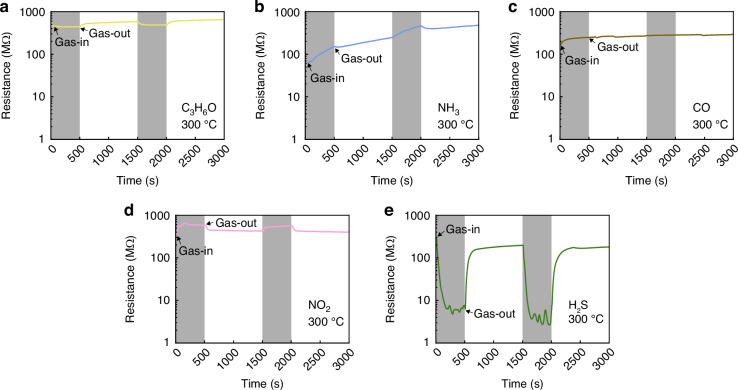
Dynamic sensing curves for different gases at 300 °C in SnO_x_-CuO_x_ solid-solution samples. **a** C_3_H_6_O, **b** NH_3_, **c** CO, **d** NO_2_, and **e** H_2_S

**Fig. 5:**
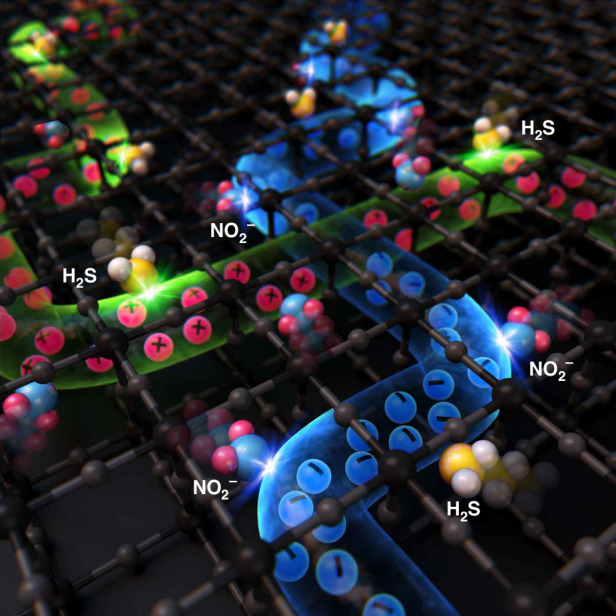
The concept of automatic selection in p- or n-type channels. Schematic illustrating the activation of different carriers (electrons and holes) when different gases (oxidising NO_2_ and reducing H_2_S) are adsorbed, resulting in the formation of two channels

However, as shown in Fig. 4, even at a temperature of 300 °C, where most carriers in n-type and p-type semiconductors can be activated, the response towards C_3_H_6_O (Fig. 4a), NH_3_ (Fig. 4b), and CO (Fig. 4c) was negligible. Furthermore, the sensing indices (e.g., response, response time, and recovery time) towards NO_2_ (Fig. 4d) decreased at this temperature. However, the behaviour toward H_2_S (Fig. 5e) completely changed from p-type to n-type at this temperature. The main cause for poor gas sensing at high process temperatures is that metal components of Sn and Cu are present alongside SnO_x_ and CuO_x_ upon formation of the two channels by FCVD; metallic properties become more prominent, and the number of activated carriers decreases at elevated process temperatures^81,82^. The SnO_x_-CuO_x_ solid-solution sample after FCVD showed a significant change in initial resistance simply because the sensing temperature had changed without any chemical reaction before the adsorption reaction with the target gas. To clarify, the unit of resistance itself changed significantly, from several kilo-ohms at room temperature and 100 °C to the mega-ohm range at 300 °C, which indicates a significant change within the sample (SI, Fig. S6a). This implies that the sample, which possesses semiconductor properties at room temperature and low temperatures, does not maintain these properties as its resistance decreases at the high temperature of 300 °C; rather, it attains metallic properties. This is because the mobile carriers (electrons and holes) in the SnO_x_-CuO_x_ solid-solution sample, which exhibits a non-equilibrium composition after the FCVD process, remain inside various defects (e.g. electron-lattice defects and oxygen vacancies) or at different impurity levels (e.g. donor level for n-type SnO_x_ and acceptor level for p-type CuO_x_) within the sample at temperatures below 100 °C. Therefore, carrier movement is suppressed, and the sample displays semiconductor properties. However, at temperatures above 300 °C, the mobile carriers are released from their binding force and delocalized; thus, the sample may exhibit metallic properties, whereby its resistance increases due to phonon scattering (SI, Fig. S6b). To verify this interpretation, the response to 10 ppm NO_2_ and H_2_S gases at RT, 100 °C, 150 °C, 200 °C, 250 °C, and 300 °C was measured, as shown in SI, Fig. S7. Except for the behaviour of H_2_S gas, which changed from p-type to n-type at 300 °C (Fig. 4e), both gases showed the highest response at 100 °C as a two-channel, while the performance decreased at RT, 150 °C, 200 °C, 250 °C, and 300 °C. Therefore, SnO_x_-CuO_x_ exhibits semiconducting properties even though there are few carriers at low temperature (100 °C) and reacts only with representative oxidising (NO_2_) and reducing gases (H_2_S).

### Carrier transport mechanism and role of the SnO_x_ and CuO_x_ junctions

In fact, this sample may be partially a p-n heterojunction, but it is entirely a solid-solution, so it may be precisely an intermediate state. Therefore, the gas sensing mechanism can be interpreted primarily as a p-n heterojunction case. From the gas sensing results above, despite the presence of different types of gases (e.g., oxidising NO_2_ and reducing H_2_S), the fact that all resistance is high indicates that different carriers (electrons or holes) contribute at the sample surface. If the sample was a single material, the NO_2_ oxidising gas and H_2_S reducing gas would have resulted in the opposite trend in resistance. In order to determine how n-type and p-type semiconductor channels exist simultaneously, the transfer path of energy (e.g., each carrier) resulting from the formation of the SnO_x_ and CuO_x_ junction by FCVD was analysed.

In this study, the oxidising gas, NO_2_, and the reducing gas, H_2_S, underwent the following chemical reactions^83,84^:

1 $${\rm{N}}{{\rm{O}}}_{2({\rm{gas}})}+{{\rm{e}}}^{-}\to {{\rm{N}}{{\rm{O}}}^{2-}}_{({\rm{ads}})}$$

2 $${\rm{N}}{{\rm{O}}}_{2({\rm{gas}})}+{{\rm{e}}}^{-}\to {\rm{N}}{{\rm{O}}}_{({\rm{gas}})}+{{{\rm{O}}}^{-}}_{({\rm{ads}})}$$

3 $${\rm{N}}{{\rm{O}}}_{2({\rm{gas}})}+{{{{\rm{O}}}_{2}}^{-}}_{({\rm{ads}})}+2{{\rm{e}}}^{-}\to {\rm{N}}{{{{\rm{O}}}_{2}}^{-}}_{({\rm{ads}})}+2{{{\rm{O}}}^{-}}_{({\rm{ads}})}$$

4 $${\rm{N}}{{{{\rm{O}}}_{2}}^{-}}_{({\rm{ads}})}+2{{{\rm{O}}}^{-}}_{({\rm{ads}})}+{{\rm{e}}}^{-}\to {\rm{N}}{{\rm{O}}}_{2({\rm{gas}})}+2{{{\rm{O}}}^{2-}}_{({\rm{ads}})}$$

5 $$2{{\rm{H}}}_{2}{{\rm{S}}}_{({\rm{g}}{\rm{as}})}+3{{{{\rm{O}}}_{2}}^{-}}_{({\rm{ads}})}\to 2{{\rm{H}}}_{2}{{\rm{O}}}_{({\rm{g}}{\rm{as}})}+2{\rm{S}}{{\rm{O}}}_{2({\rm{g}}{\rm{as}})}+{3{\rm{e}}}^{-}$$

6 $${{\rm{H}}}_{2}{{\rm{S}}}_{({\rm{g}}{\rm{as}})}+{\rm{Cu}}{{\rm{O}}}_{({\rm{s}}{\rm{ol}})}\to {\rm{Cu}}{{\rm{S}}}_{({\rm{s}}{\rm{ol}})}+{{\rm{H}}}_{2}{{\rm{O}}}_{({\rm{g}}{\rm{as}})}$$

Importantly, as already reported in our previous study^85^, the bonding energies of NO_2_ gas and H_2_S are 305.0 kJ/mol and 381.0 kJ/mol, respectively, which are the lowest among all the gases presented in Fig. 4. These low bonding energies are advantageous for gas sensing with an electrical charge. Originally, the existing PN junction was treated as the main channel through which the carrier penetrated the depleted region^86,87^. However, as shown in Fig. 5, the PN junction synthesised in this study is completely different from the conventional role through which the carrier passes.

The movement of mobile carriers within SnO_x_-CuO_x_ after the FCVD process is affected largely by two factors.

First, the 1 V voltage applied to the sample before the target gases are adsorbed may have an effect, based on which the initial resistance of the sample is determined. At this point, due to FCVD, non-equilibrium semiconductors of various compositions, such as SnO_x_ and CuO_x_, are formed inside the sample. The representative semiconductor compositions are as follows: SnO_2(n-type)_, SnO_(p-type)_, Cu_2_O_(p-type)_, and CuO_(p-type)_. Most of these, except SnO_2_, are p-type semiconductors^88–91^. Therefore, when a voltage of 1 V is applied, the movement of electrons is inevitably affected by the depletion layer of various PN junctions, whether in the forward or the reverse direction, as shown below:

7 $${\rm{Sn}}{{\rm{O}}}_{2({\rm{n}}-{\rm{type}})}\to {\rm{Sn}}{{\rm{O}}}_{({\rm{p}}-{\rm{type}})}$$

8 $${\rm{Sn}}{{\rm{O}}}_{2({\rm{n}}-{\rm{type}})}\to {\rm{Sn}}{{\rm{O}}}_{({\rm{p}}-{\rm{type}})}\to {\rm{Cu}}{{\rm{O}}}_{({\rm{p}}-{\rm{type}})}$$

9 $$\mathrm{Sn}{{\rm{O}}}_{2({\rm{n}}-\mathrm{type})}\to \mathrm{Sn}{{\rm{O}}}_{({\rm{p}}-\mathrm{type})}\to \mathrm{Cu}{{\rm{O}}}_{({\rm{p}}-\mathrm{type})}\to {\mathrm{Cu}}_{2}{{\rm{O}}}_{({\rm{p}}-\mathrm{type})}$$

However, when a voltage of 1 V is applied, holes do not necessarily have to pass through the depletion layer of the PN junction, as expressed below. This means that movement is possible only through the PP junction.

10 $$\mathrm{Cu}{{\rm{O}}}_{({\rm{p}}-\mathrm{type})}\to {\mathrm{Cu}}_{2}{{\rm{O}}}_{({\rm{p}}-\mathrm{type})}\to \mathrm{Sn}{{\rm{O}}}_{({\rm{p}}-\mathrm{type})}$$

The reason for this difference in electron and hole movement mechanisms is that p-type materials, such as SnO, are formed from SnO_2_ through the FCVD process. Therefore, since no special n-type channel, except for the SnO_2_ channel, is present on the electron side, it must pass through the PN depletion layer. However, on the hole side, several p-type channels, such as CuO, Cu_2_O, and SnO, are present; therefore, the PN depletion layer can be bypassed. Thus, due to the applied voltage, the PN depletion layer becomes more involved in the SnO_x_ channel.

Second, changes may occur in the PN junction after the target gases are adsorbed. For example, as seen in SI, Table S2, the response of NO_2_ (16.7 at 10 ppm and 100 °C) is about 10 times that of H_2_S (1.68 at 10 ppm and 100 °C), while the response time of NO_2_ (126 s at 10 ppm and 100 °C) is about twice as long as that of H_2_S (91 s at 10 ppm and 100 °C). This is because the electron concentration at the NN junction decreases with the introduction of the NO_2_ target gas and because the depletion layer at the PN junction formed inside widens, increasing the resistance. Consequently, the response is high, and the response time is long, because the flow of electrons is impeded. Conversely, the introduction of the H_2_S target gas can reduce the hole concentration at the PP junction. Instead, however, since the hole can bypass the PN junction, the change in resistance is not significant; hence, the response is low, and the response time is shortened.

As shown in Fig. 1, the FCVD process causes many morphological, compositional, crystallographic, and electrical changes in the initial Cu and SnO_2_ bilayer. Morphologically, bumpy SnO_x_ and CuO_x_ are observed (Fig. 1c). Compositionally, the SnO_2_ side exhibited compositions with various proportions of Sn and O, ranging from SnO_2_ to Sn, while the Cu side simultaneously displayed various compositions, ranging from Cu to CuO (Fig. 1d–f). Crystallographically, various non-equilibrium phases existed, which were somewhat different from those indicated by the initial equilibrium peaks of SnO_2_ and Cu (Fig. 1h). Electrically, as seen in Fig. 3, the resistance increased for both NO_2_, an oxidising gas, and H_2_S, a reducing gas, which possess opposite properties. This implies that both electron and hole channels can be involved, as shown in Fig. 5. Essentially, the SnO_x_-CuO_x_ solid-solution sample exhibits n-type characteristics, as suggested by the relationship between the valence band maximum, Fermi level, and work function in SI, Fig. S3. Therefore, for NO_2_ gas, electron carriers are involved, considering its n-type characteristic. However, for H_2_S gas, hole carriers are involved, which is opposite to the n-type characteristic. The main reason for these differences is the difference in gas sensitivity: energetically, it is easier for the oxidising gas to accept electrons from the electron-rich SnO_x_, whereas it is easier for the reducing gas to fill holes in CuO_x_ with the supplied electrons. However, as mentioned before, in the case of electron movement, the depletion layer of the PN junction formed inside must also be passed through; therefore, the resistance increases, and the response time is also lengthened. Conversely, in the case of hole movement, the resistance decreases, and the response time becomes shorter, because the increase in the amount of p-type compounds, such as SnO, CuO, and Cu_2_O, allows for a bypass to the PN junction.

**Fig. 6:**
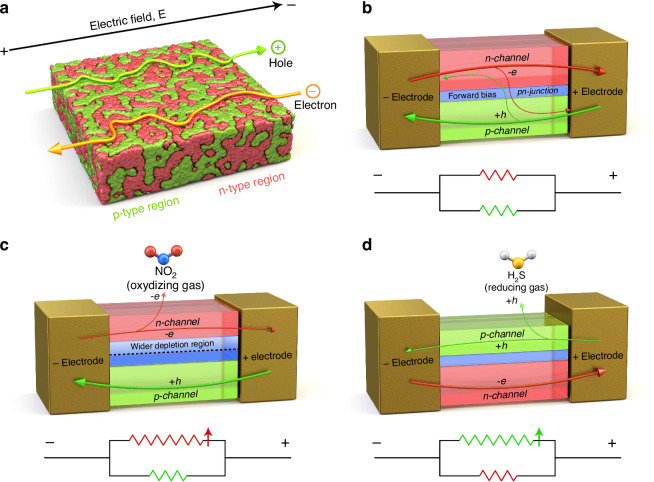
Schematic showing the mechanism behind the formation of NN, PP, and PN junctions in SnO_x_-CuO_x_ solid-solution samples following FCVD. **a** NN and PP junctions formed in three dimensions. **b** Types of channels formed at the electrodes and the direction of mobile carriers. **c** Changes in NN channel, PN junction, and resistance when NO_2_ gas is adsorbed. **d** Changes in PP channel and resistance when H_2_S gas is adsorbed

After the FCVD process, the domains of SnO_x_ and CuO_x_ can be connected to each other while being entangled in three dimensions, as shown in Fig. 6a. In other words, NN, PP, and PN junctions are uniformly connected to each other. Even if minority carriers exist, each junction acts as a resistive element during gas sensing because it impedes the movement of carriers (Fig. 6b). Moreover, mobile carriers tend to flow through the path of least resistance. Figure 6c and d depict the movement of electrons and holes. First, when NO_2_ gas is adsorbed, it accepts electrons from the sample, reducing the number of electrons in the SnO_x_ channel; this ultimately widens the depletion layer of the PN junction and narrows the conductive channel, thereby increasing the resistance (Fig. 6c). Meanwhile, when H_2_S gas is adsorbed, it supplies electrons to the sample, reducing the number of holes in the CuO_x_ channel; thus, the conductive channel is ultimately narrowed, while the width of the PP junction is reduced; this again increases the resistance (Fig. 6d). This sensing mechanism can be approximated by a resistive circuit with two main conduction paths—an NN + PN channel and a PP channel—connected in parallel (Fig. 6b–d). Interactions between the sample and the target gas selectively increase the resistance of one of the two paths (Fig. 6c and d). In a parallel circuit, if one resistance increases significantly, the total equivalent resistance also increases. Therefore, the overall resistance of the gas sensor can be increased for two different types of gases. However, inferring these charge transport pathways cannot be considered direct evidence. This is because the SnO_x_-CuO_x_ sample obtained by the FCVD method has both p-n heterojunction and solid-solution characteristics as mentioned above, so interpretation of only one side is not perfect. However, from a solid-solution perspective, the same results can be obtained as follows.

Within this solid-solution, Sn- and Cu-related sites coexist at the nanoscopic scale, giving rise to spatially distributed electron- and hole-sensitive regions. Oxidising NO_2_ and reducing H_2_S preferentially interact with different local sites; however, both gas species ultimately increase carrier depletion and potential fluctuations within the solid-solution matrix. As a result, charge transport through the SnO_x_-CuO_x_ solid-solution becomes increasingly barrier-limited, leading to resistance-increasing responses for both gases.

For this reason, the potential for these concepts is large, so it is necessary to continuously deepen our understanding of the complete mechanism and continue to study the same sample series in the future. Furthermore, continued use of FCVD will be investigated for the development of multi-functional materials and devices.

## Conclusions

By utilising a relatively simple surface modification strategy called FCVD, a nanocomposite of SnO_2_ and Cu bilayers was synthesized, providing a new material in which SnO_x_ and CuO_x_ were uniformly distributed. Due to the combination of n-type and p-type non-equilibrium metal oxides, a unique structure was obtained in which a single sample could perform two opposing functions on the material surface. This was confirmed by analysing both the entire sample and localised regions through constituent analysis, especially when an oxidising gas (e.g. NO_2_) or reducing gas (e.g. H_2_S) was adsorbed onto the sample surface, resulting in the same pattern in change in resistance rather than the opposite. For example, in a NO_2_ gas concentration of 10 ppm at 100 °C, n-type SnO_x_ was activated and showed a response of 16.7. On the other hand, in a H_2_S gas concentration of 10 ppm at 100 °C, p-type CuO_x_ was activated and showed a response of 1.99. Ultimately, the material behaved as if selecting a favourable channel on its own, depending on the type of gas. A technique capable of simultaneously realising p- and n-type semiconductors in one sample eliminates many of the problems that may arise from their consistency during the manufacturing process of nano devices, and this technique will certainly be an important starting point for exponentially improving its efficiency and speed.

## Supplementary information

Supplementary Information

## Data Availability

All the data are available from the corresponding author upon reasonable request.
