# Supplementary material for: A synthetic method for preparing double channelling materials, and an operational mechanism for selective p- and n-type channels for gas sensing
Source: Microsyst Nanoeng. 2026 May 6;12:160. doi: 10.1038/s41378-026-01253-w (PMC13144517; doi:10.1038/s41378-026-01253-w)
Supplement: Supplementary file 1 — Supplementary Information [file 41378_2026_1253_MOESM1_ESM.docx]

**Supporting Information**

**A synthetic method for preparing double channelling materials, and an operational mechanism for selective p- and n-type channels for gas sensing**

Myung Sik Choi^1^, Han Gil Na^2^, Jeong Yun Hwang^3^, Seung Yong Lee^4^, Sanghyun Ji^3^, Jimyeong Park^3^, Sun-Woo Choi^5^, Kyu Hyoung Lee^3,*^ & Changhyun Jin^3,*^

^1^Department of Nano & Advanced Materials Science and Engineering, Kyungpook National University, Sangju 37224, Republic of Korea

^2^UDerive, Incheon 22824, Republic of Korea

^3^Department of Materials Science and Engineering, Yonsei University, Seoul, 03722, Republic of Korea

^4^Division of Advanced Materials Engineering Kongju National University Cheonan, 31080, Republic of Korea

^5^Department of Materials Science and Engineering, Kangwon National University, Samcheok 25913, Republic of Korea

Myung Sik Choi and Han Gil Na had equal contribution as co-first authors.

*Correspondence to: khlee2018@yonsei.ac.kr (K.H. Lee), z8015026@yonsei.ac.kr (C. Jin)

**
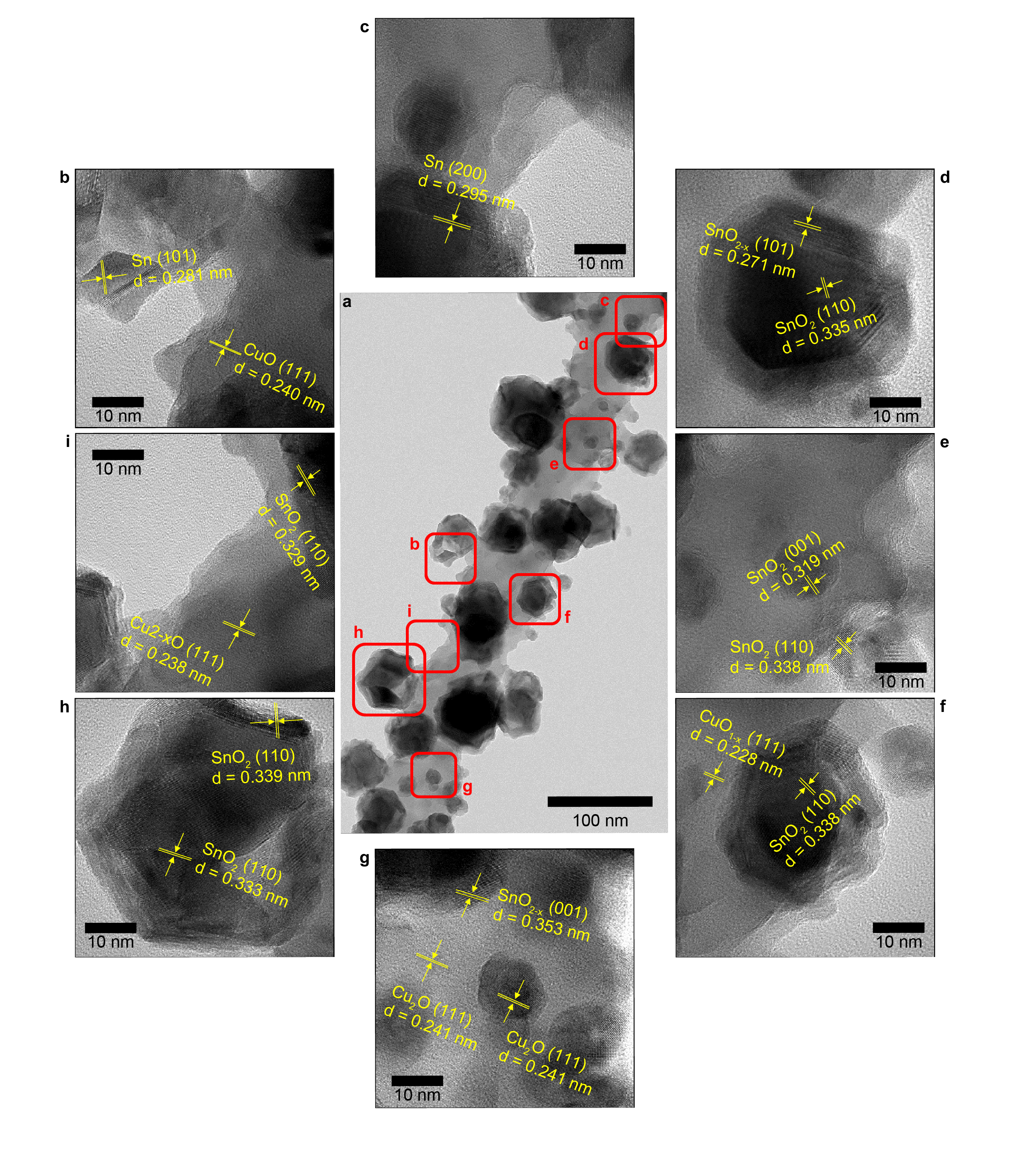
**

**Fig. S1** (a) TEM and (b–i) HRTEM images showing different morphologies and compositions of the (a) whole and (b–i) regions of the SnO_x_-CuO_x_ solid-solution formed by FCVD.


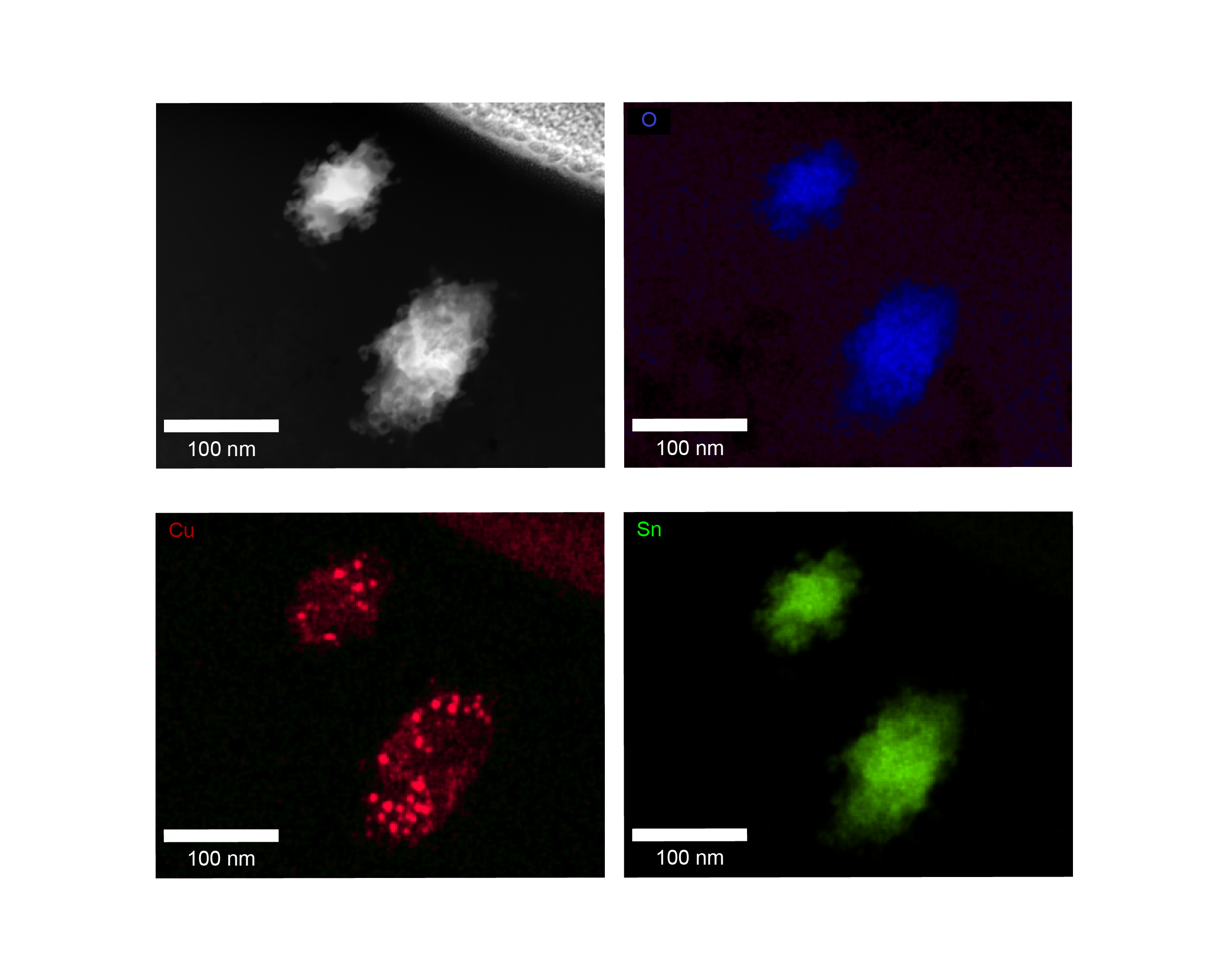


**Fig. S2** Cu, Sn, and O compositions detected by HAADF.


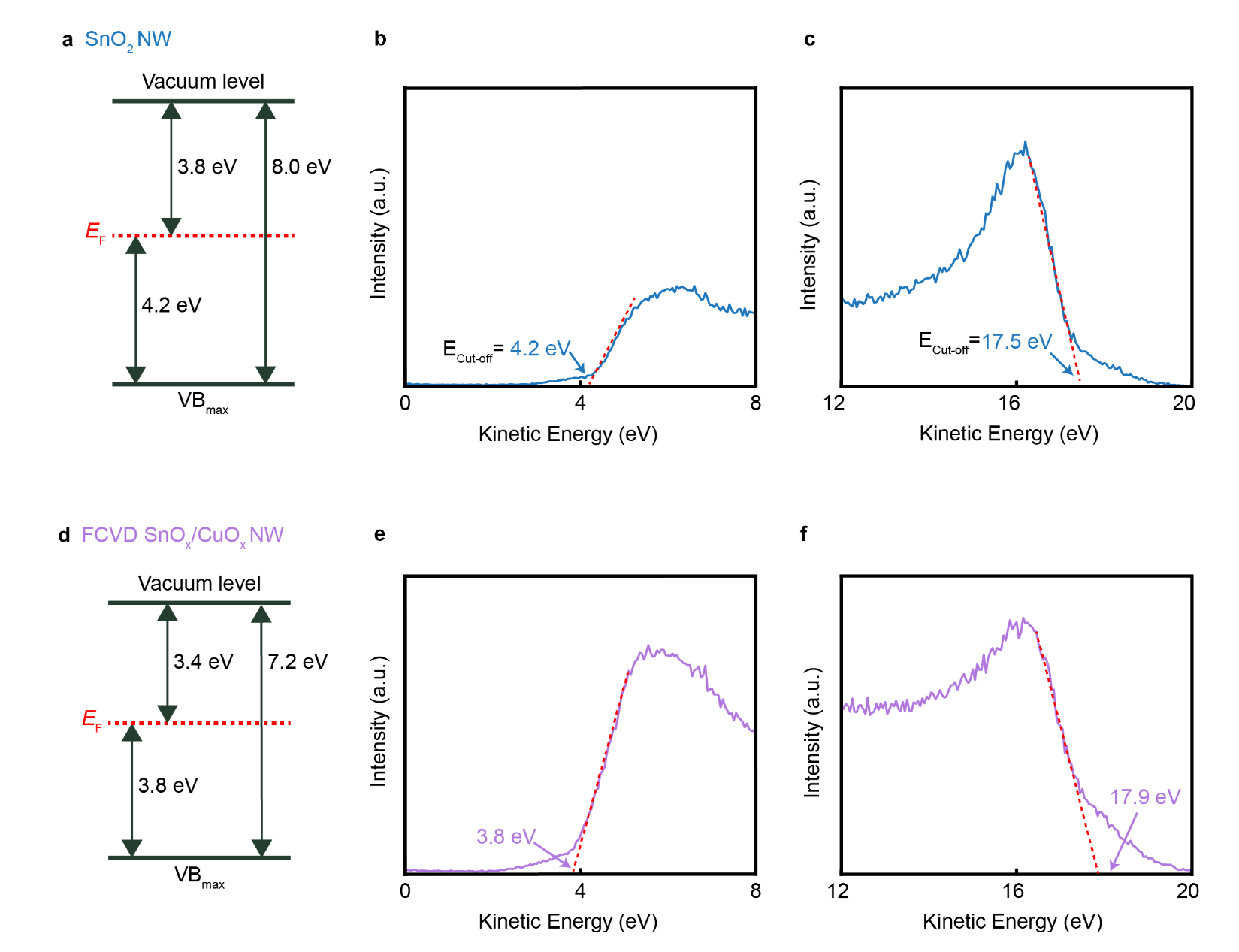


**Fig. S3** Electrical properties of the (a–c) bare SnO_2_ nanowires and (d–f) SnO_x_-CuO_x_ solid-solution nanowires. (a) Relationship among the valence band maximum, Fermi level, and work function, and (b–c) relationship between the kinetic energy and intensity of the bare SnO_2_ nanowires. (d) Relationship among the valence band maximum, Fermi level, and work function, and (e–f) relationship between the kinetic energy and intensity of the SnO_x_-CuO_x_ solid-solution nanowires.


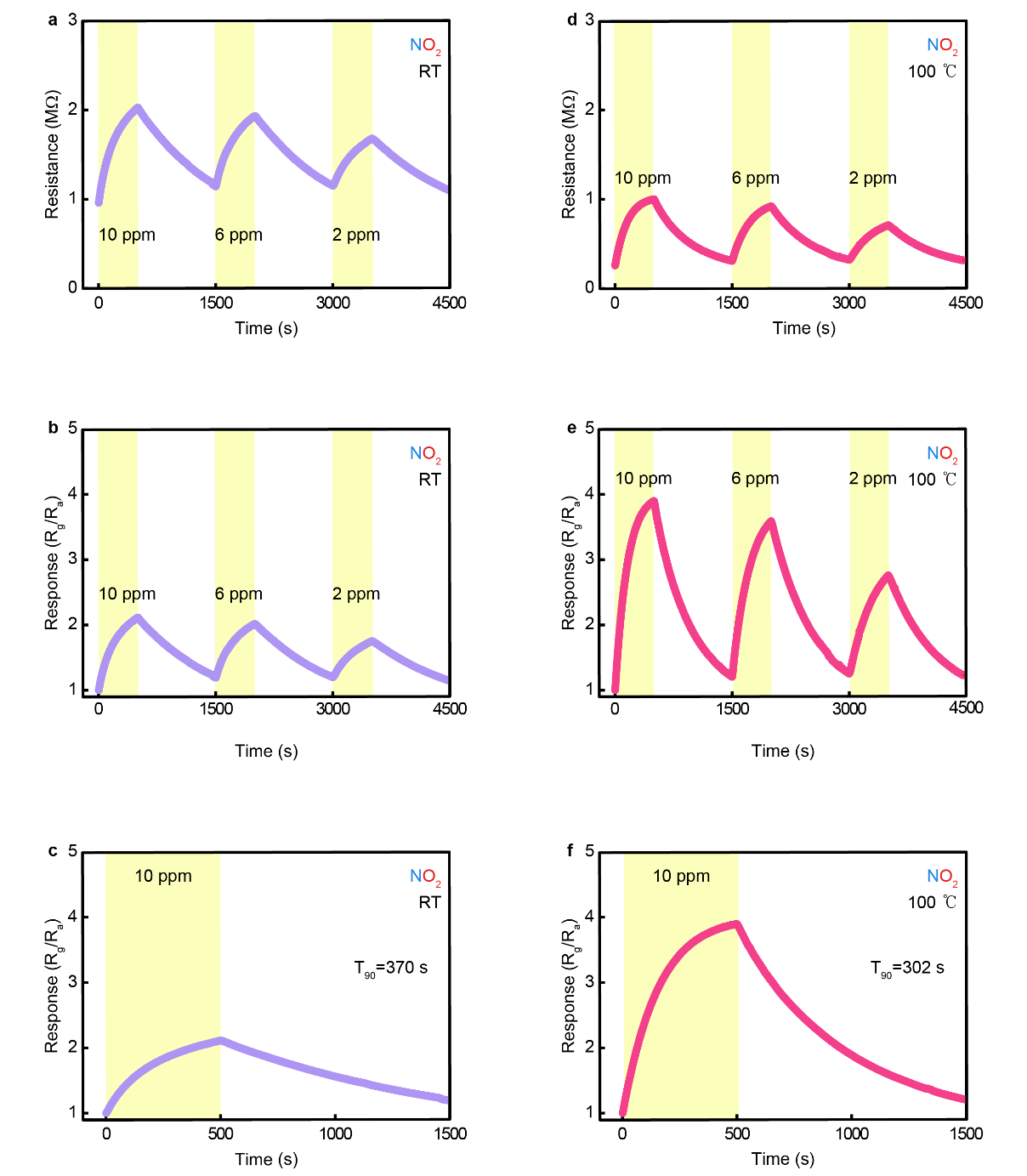


**Fig. S4** Gas sensing responses at RT (25°C) and 100°C for 2, 6, and 10 ppm NO_2_ of bare SnO_2_. (a–c) RT, (d–f) 100°C.

**
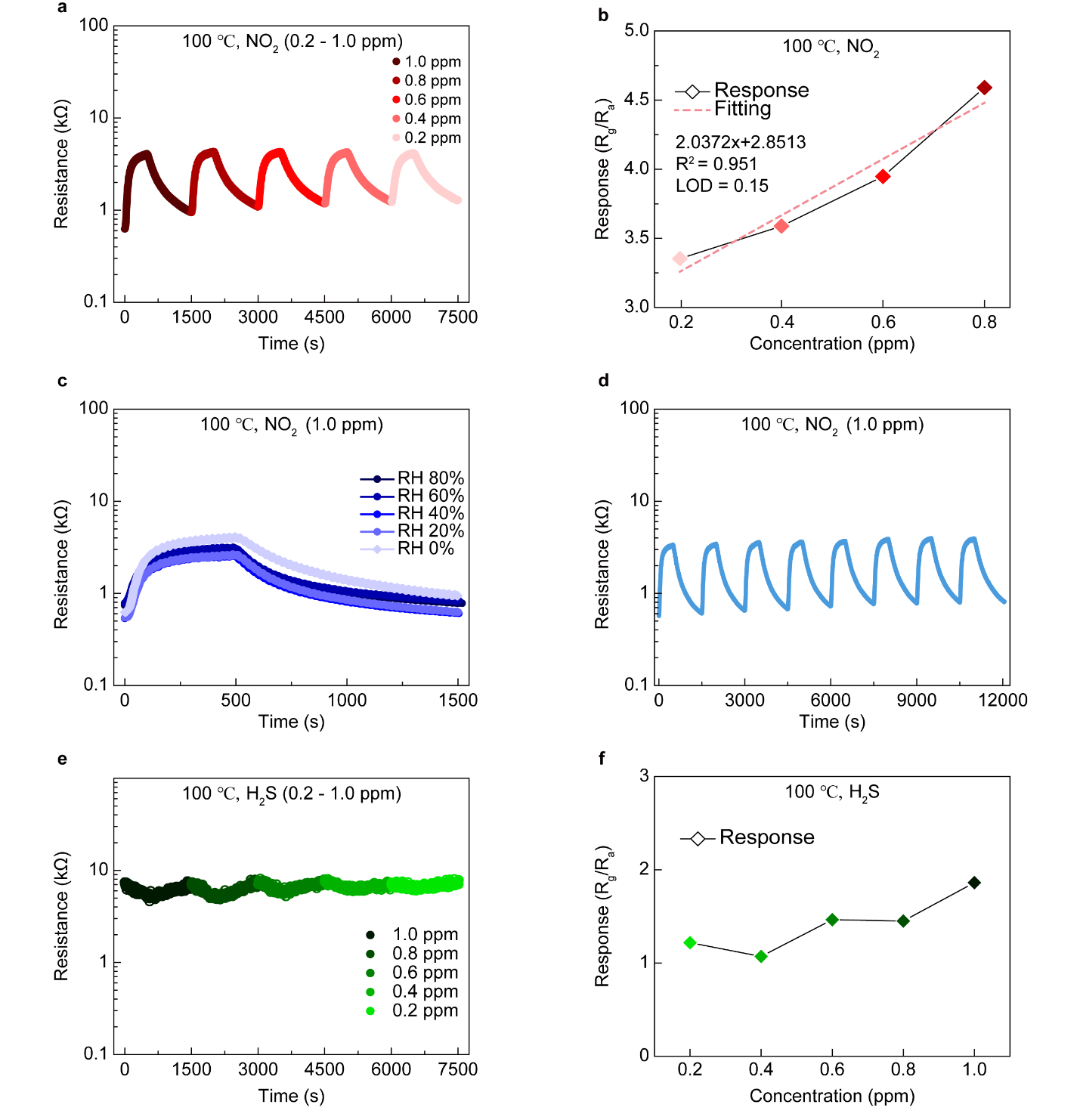
**

**Fig. S5.** Stability evaluation of SnO_x_-CuO_x_ solid-solution gas sensors at 100°C. (a-d) NO_2_ gas, (a) low gas concentrations, (b) limit of detection (LOD), (c) humidity, (d) repeatability, (e-f) H_2_S gas, (e) low gas concentrations, (f) responses according to gas concentrations.


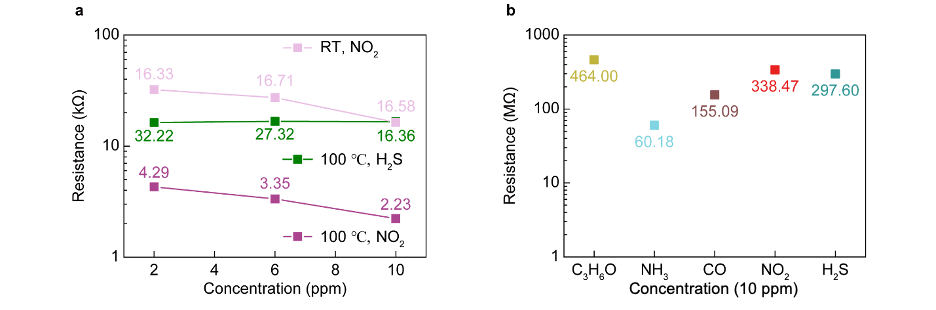


**Fig. S6.** Changes in the initial resistance of various target gases with temperature in a SnO_x_-CuO_x_ solid-solution. (a) RT and 100°C, (b) 300°C

**
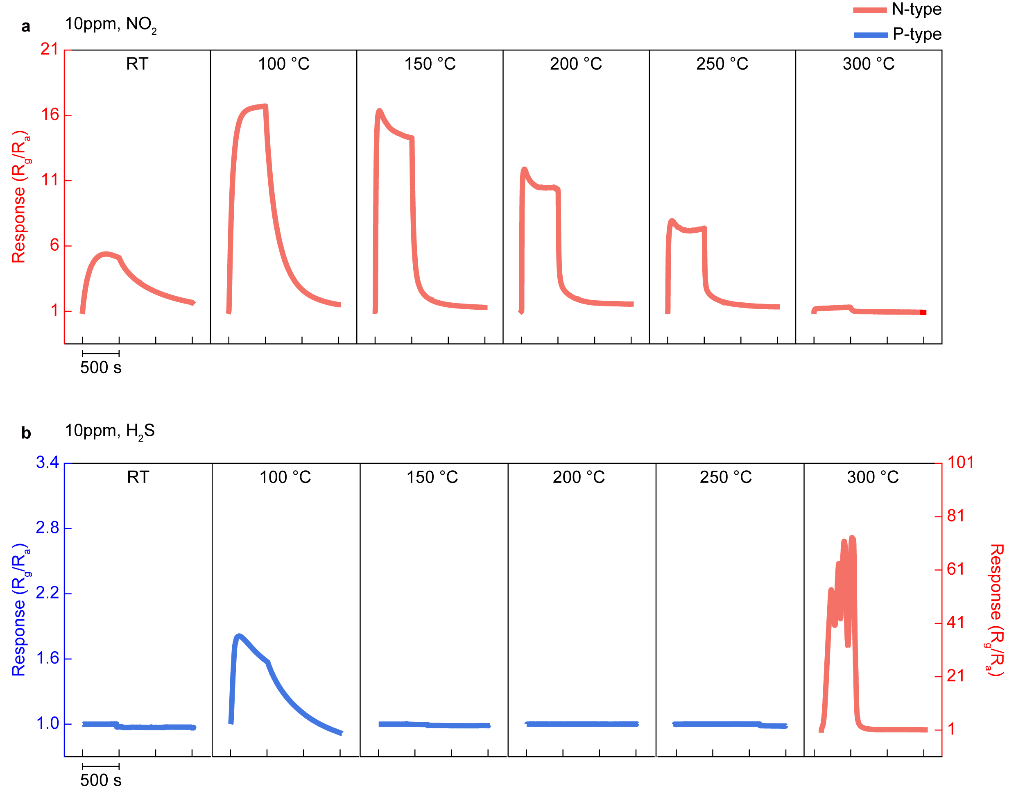
**

**Fig. S7.** Comparison of responses to 10 ppm (a) NO_2_ gas and (b) H_2_S gas in SnO_x_-CuO_x_ solid-solution samples at RT, 100°C, 150°C, 200°C, 250°C, and 300°C.

**Table S1**. Comparison of sample characteristics before and after the FCVD process.

|  | **Before FCVD** | **After FCVD** |
| --- | --- | --- |
| Synthesis method | Thermal evaporation and sputtering | Flame chemical vapor deposition |
| Synthesis time | > 1 hour | 5 seconds |
| Pre- and post-treatment | Catalyst needed | N/A |
| Vacuum | 2 x 10^–4^ mTorr | N/A |
| Composition | Equilibrium phases | Non-equilibrium phases |
| Structure | Double structure of SnO_2_ and Cu | Solid-solution structure of SnO_x_-CuO_x_ |
| Surface defects | Minority | Majority |
| Adsorption surface of target gas | Cu layer | SnO_x_ and CuO_x_ |
| Semiconductor Channel | N/A | Two channels |
| Gas sensing | Possible | Impossible |

**Table S2**. Sensing responses exhibited by the SnO_x_-CuO_x_ solid-solution.

|  | **NO_2_ gas at 100℃** | | | **H_2_S gas at 100℃** | | | **NO_2_ gas at RT (25°C)** | | |
| --- | --- | --- | --- | --- | --- | --- | --- | --- | --- |
| Concentration  (ppm) | 2 | 6 | 10 | 2 | 6 | 10 | 2 | 6 | 10 |
| Response  (R_g_/R_a_) | 13.96 | 16.62 | 16.7 | 1.55 | 1.68 | 1.99 | 3.82 | 4.47 | 5.38 |
| Response time  (s) | 261 | 194 | 126 | 224 | 91 | 59 | 387 | 311 | 171 |

**Table S3.** Comparison of proposed process with previously reported techniques based on SnO₂/CuO heterojunctions.

| Material | Optimized temperature (°C) | Response to NO_2_ (Conc.) | Response to H_2_S | Ref. |
| --- | --- | --- | --- | --- |
| CuO_x_-SnO_x_ NWs FCVD | 100 | 16.7 (10 ppm) | 5.38 (10 ppm) | **This work** |
| CuO/SnO_2_ | 150 | 56 (1 ppm) | - | (S1) |
| SnO_2_/CuO nanohydrangeas | 100 | 159 (5 ppm) | - | (S2) |
| Cu-loaded SnO_2_ nanoparitcles | 200 | 5680 (5 ppm) |  | (S3) |
| SnO_2_-CuO nanocomposite | 100 | - | 4173 (10 ppm) | (S4) |
| CuO-SnO_2_ | 200 | - | 7341 (20 ppm) | (S5) |
| CuO-loaded SnO_2_ hollow spheres | 300 | - | 22.4 (1 ppm) | (S6) |
| CuO-loaded SnO_2_ nanofibers | 300 | - | 1.25$\times$10^4^ (1 ppm)  1.98$\times$10^4^ (1 ppm) | (S7) |
| Porous SnO_2_-CuO nanotube | 150 | - | 45,988 (40 ppm) | (S8) |
| CuO-doped SnO_2_ NWs | 300 | - | 809 (20 ppm) | (S9) |
| CuO-doped SnO_2_ nanoparticles | 125 | - | 1056 (10 ppm) | (S10) |

[S1] Shi, Z.-H., Hsiao, Y.-J., Chang, S.-P. & Chang, S.-J. Synthesis and characterization of an oxygen-controlled CuO/SnO_2_ sensor for NO_2_ detection. *Sens. Actuator B-Chem.* **421**, 136517, (2024).

[S2] Shi, Z.-H., Hsiao, Y.-J., Chang, S.-P. & Chang, S.-J. Synthesis and characterization of an oxygen-controlled CuO/SnO_2_ sensor for NO_2_ detection. Sens. Actuator B-Chem. 421, 136517, (2024).

[S3] Li, S., Li, X., Guo, J., Liu, X. & Zhang, J. Heterostructured SnO_2_/CuO Hydrangea Nanocomposites for Highly Sensitive NO_2_ Detection. *ACS Appl. Nano Mater.* **8**, 10549-10558, (2025).

[S4] Leangtanom, P. *et al.* Enhanced NO_2_-Sensing Properties of Cu-Loaded SnO_2_ Nanoparticles Synthesized via Precipitation and Impregnation Methods. *Phys. Status Solidi A-Appl. Mat.* **219**, 2100797, (2022).

[S20] Gao, C., Lin, Z.-D., Li, N., Fu, P. & Wang, X.-H. Preparation and H_2_S Gas-Sensing Performances of Coral-Like SnO_2_–CuO Nanocomposite. *Acta Metall. Sin.-Engl. Lett.* **28**, 1190-1197, (2015).

[S5] Chowdhuri, A., Sharma, P., Gupta, V., Sreenivas, K. & Rao, K. V. H2S gas sensing mechanism of SnO_2_ films with ultrathin CuO dotted islands. *J. Appl. Phys.* **92**, 2172-2180, (2002).

[S6] Choi, K.-I., Kim, H.-J., Kang, Y. C. & Lee, J.-H. Ultraselective and ultrasensitive detection of H_2_S in highly humid atmosphere using CuO-loaded SnO_2_ hollow spheres for real-time diagnosis of halitosis. *Sens. Actuator B-Chem.* **194**, 371-376, (2014).

[S7] Choi, S.-W., Zhang, J., Akash, K. & Kim, S. S. H_2_S sensing performance of electrospun CuO-loaded SnO_2_ nanofibers. *Sens. Actuator B-Chem.* **169**, 54-60, (2012).

[S8] Park, K.-R. *et al.* Design of highly porous SnO_2_-CuO nanotubes for enhancing H_2_S gas sensor performance. *Sens. Actuator B-Chem.* **302**, 127179, (2020).

[S9] Hwang, I.-S. *et al.* Enhanced H_2_S sensing characteristics of SnO_2_ nanowires functionalized with CuO. *Sens. Actuator B-Chem.* **142**, 105-110, (2009).

[S10] Chen, Z., Xu, Z. & Zhao, H. Flame spray pyrolysis synthesis and H_2_S sensing properties of CuO-doped SnO_2_ nanoparticles. *Proc. Combust. Inst.* **38**, 6743-6751, (2021).
